# Peasants and migrant workers in the farms and the mines: synergies and contradictions

**DOI:** 10.1007/s10460-025-10719-y

**Published:** 2025-03-25

**Authors:** Doi Ra

**Affiliations:** International Institute of Social Studies of Erasmus University Rotterdam, Den Haag, Netherlands

**Keywords:** Land rush, Migrant labour, Social reproduction, Peasants, Agriculture, Mine workers, Mining, Kachin, Myanmar

## Abstract

This paper builds on a reflection by Nicholson, a founding member of La Via Campesina, on the contradictions faced by peasant households worldwide—specifically, the increasing necessity to combine wage work with small-scale farming and the use of migrant labour. For peasant households, contradiction exists not only in farming but also in relation to other sectors where they themselves engage in wage work, such as in the agribusiness, service sector or in the mines. Focusing on the aspects of contradictions, this paper explores the situation of peasant households and migrant mine workers in Kachin State, Myanmar, as they navigate a socio-ecological landscape transformed by land rushes. The processes driving the mining economy are intertwined with and, in some ways, support the persistence of peasant agriculture. This means that the mining industry, while is often viewed as detrimental to rural livelihoods, is paradoxically contributing to the survival of peasant households.

## Introduction

This paper builds on a reflection by Nicholson, a founding member of La Via Campesina on the current state of peasant households around the world (Nicholson and Borras 2023). He brought up a common situation among majority of the peasant households around the world today as they combine small-scale farming with different wage work to ensure continued social reproduction. In response to the situation, he reflected as followed, “This is our reality, but it does not mean that we do not have contradictions” (Nicholson and Borras 2023, p. 619). Nicholson was highlighting the contradictions among which under certain circumstances, peasant farming has to hire-in outside labour, using cheap labour of the migrant workers. For peasant^1^ households, contradiction exists not only in farming but also in relation to other sectors where they themselves engage in wage work, such as in the agribusiness, service sector or in the mines.

Exploring this dynamic, this paper examines the situation of peasant households in Kachin State, Myanmar (formerly Burma), as they navigate a socio-ecological landscape transformed by land rushes. This transformation has led to increased reliance on wage labor, particularly in the mining sector, and has created new challenges for peasant households seeking to maintain their livelihoods and social reproduction. In order to achieve the objective, the article investigates the current situation of peasant farming, traces the division and fragmentation of peasant household labour across the spatial continuum, with continued linkages to farming as a safety net or a fallback position, a cultural identity and a future aspiration (Cousins 2022; Huijsmans et al. 2021; Van Der Ploeg 2023). The article also examines the gendered and generational dimensions of struggles for social reproduction, highlighting women’s increased responsibilities in the “economies of care” (the productive work necessary to sustain the daily lives of working people and their family members) and the increasing involvement of young men in the dangerous mining sites for migrant wage work (Akram-Lodhi and Huijsmans 2024; Chazali et al. 2024; Hajjar et al. 2020; Shah and Lerche 2020).

This article argues that the reproduction of peasant households and farming is strongly linked to mining sector. Such condition has been facilitated by the land rush which has exponentially transformed rural landscapes into “fictitious commodities” (Polanyi 1944). Against the rapidly changing landscape, the article highlights an unequal synergetic relationship between the two seemingly contradictory yet historically entangled sectors– peasant agriculture and mining. Together, these sectors have addressed the social reproduction needs of the rural peasant households. Echoing previous studies, this article supports the importance of access to land for rural populations, emphasizing that without it, many would face even greater hardship (Ossome 2024). At the same time, access alone is not enough, as the Kachin case—to be elaborated later—shows. It must be accompanied by other structural and istitutions restructuring (Rigg 2006; Van Der Ploeg 2023).

This article is organized as follows. The next section briefly discusses the theoretical framework, followed a short section that explains how the data were collected. This is then followed by case studies from Kachin state to shed light on the current situation of access to land, production challenges, migration, and their social reproduction struggles. This will be followed by the findings from a household survey about livelihood strategies which the rural households engage in along the continuum of rural-urban and farm-non-farm spaces. In the next section, mining as a livelihood strategy among peasant households is discussed in order to understand the reasons behind its growing popularity as a migrant labour destination. Finally, the implications for peasant farming and rural households in shaping the landscape are discussed.

## Theoretical discussion

This study uses the context of land rush as a key analytical framework to study transformations around rural communities in Kachin state. Land rush is defined by Borras and Franco (2024, p. 3) as something that denotes “a chaotic, relatively short-lived, historical juncture marked by a sudden surge in demand for land, accompanied by an extremely speculative and competitive, often violent and convulsive transition from one set of rules on commodity and land politics to another.” The authors identify three conditions that trigger a land rush, either singularly or in combination. This article focuses on two: first, a sudden rise in speculative demand for land in the absence of a clear governance system; and second, when new rules and regulations redefine land allocation, sparking competition among actors. The main push for the land rush develops from a sense of extreme urgency and competition—to secure land as “land prospectors” or to secure land-based investments as “investment prospectors.” Hence, land rushes are characterized by short durations and an atmosphere of irrationality, often fueled by spectacle and spectacularization. This leads to land rushes being driven by a diverse range of intimate and distant actors, wild speculations, hyperbolic claims, extravagant performances, and an atmosphere of upheaval and rapid change (Borras Jr and Franco 2024; Fairbairn 2020; Tsing 2005a).

Overall, land rushes can be followed by land enclosures, land grabs, land deals, land acquisitions, and commodity booms of varying scales. The land rush ultimately has the effect of partially or completely transforming land regimes which refer to “established patterns of rules on how to govern access, use, control, and ownership of land across sectors and in the rural-urban continuum” (Borras Jr and Franco 2024, pp. 4–5). In many instances, land grab literature tends to focus on single cases demarcated by physical and sectoral boundaries within a timeline (the corporate timeline process of land acquisition, for example). It is indeed crucial in studying land deals to investigate social dynamics confined in space. However, if we are trying to understand the broader social change then such spatial or sectoral demarcation becomes insufficient. Using land rush as a key conceptual handle enables investigation of how the landscape (Mitchell 1996) of Kachin State has been transformed as a whole, enabling to move beyond siloed perspectives.

In analyzing the formation of landscapes, Mitchell (1996) emphasized the centrality of labour-capital relations in shaping the social and material dimensions as he investigated the production of California landscape based on industrialized agriculture. According to him, “a landscape is a “work”—a work of art, and worked land” (1996, p. 6). Landscape appears natural in a particular morphological form because labour was invested to make it appear that way while compelled to hide its processes. Mitchell connected to Marx’s explanation on labour, which is that the socially organized labour of people creates metabolism between humans and nature, so “the work of people (re)produces a (socialized) nature—and, perforce, it produces landscapes, it transforms the land” (1996, p. 6). For Marx (1978), the capitalist mode of production transforms social structures in ways that can alienate the fruits of labour from the labourers and the larger society. Building on Marx’s work, Mitchell (1996, p. 7) explained that the act of alienation and maintaining alienation require a lot of work. So, for him, only by attending closely to the underlying and historical social relations of labour can we understand the form and meaning of a given landscape (Mitchell 1996).

In his intervention in the global land rush literature, Carlos Oya (2013, 516) questioned, “Is land everything for the ‘poor’?” With this he sought to raise the question of whether land is the only determining factor in the lives of the impoverished rural populations when various forms of exploitation exist under capitalism. In order to engage with the challenging but urgent agrarian question of labour, especially in the context of the global south, he proposed to focus on livelihoods with “a more serious and solid approach” (Oya, 2013b, p. 1554). He suggested putting labour and exploitation as central analytical and empirical categories to address many of the unresolved issues within the global land grab debate (Oya, 2013a). To follow Oya’s direction, the discussions taken by Li (2011) and Edelman et al. (2013) are used to nuance analytical capability. They center labour in the land grab literature by presenting two scenarios: (1) when the land is needed but the labour is not, and (2) when capital needs both land and labour. Both scenarios can be linked to increased social reproduction crisis and dispossession of peasants and middle farmers in the countryside (Li, 2011). When the land is needed but the labour is not, what happens to the affected local communities? As succinctly put by Li (2011, pp. 294–296), they are branded with a “de facto proletarian status without a proletarian future” followed by the question “… where will these people go, and what will they do”, and as phrased by Scott (1976, p. 7), “what is left?” from the perspective of moral economy of the “subsistence ethic”.

On the other hand, agrarian communities lose their ability to reproduce themselves outside commodity relations and markets without necessarily being dispossessed of their land (and other means of production) (Bernstein 2010, p. 34) through relations and processes of adverse incorporation (Hall, 2011; Hickey & du Toit, 2013). The arrangements of subsumption by capital vary in many forms such as wage work (Habibi & Juliawan, 2018), through different production arrangements such as contract farming (Hall et al. 2017; Oya, 2012) or through climate change mitigation schemes such as REDD+ (Corbera, 2012). This process has been termed “semi-proletarianization” of small and marginal peasant producers (Arrighi, 1970; Kautsky, 1988; Wolpe, 1972) in which peasants are not physically dispossessed from their lands but become wage labourers on their own land or must combine wage work with farming for survival. Access to land for farming and other purposes subsidized the cost of social reproduction allowing “super-exploitation by capital” (Levien et al., 2018, p. 868). The struggles for social reproduction shape the strategies of rural households for survival, which in turn affect the social trajectories of these households (Cousins 2022). Yet, the underlying catalyst for change remains the “restless and relentless forces of capital in one guise or another (ibid.).

While different combinations of relationships between land and labour may result in varying social trajectories, this article supports access to land as crucial for the rural poor, if not an absolute necessity in the context of Myanmar. Land and agriculture play crucial roles in the lives and livelihoods of rural peasant households, including those who are engaging in wage work away from their homes but retain their rural-rootedness (Borras 2023; Cousins 2022). Over time, the significance of land for agriculture has relatively declined compared to other sectors, reflected in the decreasing share of agriculture in the Gross Domestic Product (GDP) of many countries (World Bank, n.d.). But even as the rural populations become less reliant on land for farming as the main income source, land remains central to their social reproduction and productive activities. This is demonstrated by a recent study on Myanmar’s rurally-rooted cross-border migrant workers (Borras et al. 2022). In practical terms, land provides a means of subsistence, acts as a shock absorber of external risks, provides access to other natural resources such as water and forests, stores biodiversity and ecological services, and for many social groups, it carries cultural and religious meanings. Further exploration of land’s significance as a space for social reproduction is still needed, encompassing meanings and purposes beyond the narrow notion of economic production often emphasized in critical agrarian studies (Zhan and Scully 2018).

In this article, the terms “peasants” (Edelman, 2013) and “working people” (Shivji 2017) are used based on the shared similarities applicable to the social group of interest. This study adopts Edelman’s (2013) synthesis of “peasant” as a diverse group engaged in small-scale farming alongside various other livelihood strategies. These include wage labor, livestock production, artisanal mining, fishing, foraging, petty trade, and other skilled and unskilled occupations. Similar to Via Campesina, which use “peasant” as a political category signifying “people of the land” to foster broader coalitions, Edelman identifies key political characteristics associated with the term. These include facing discrimination through legal and extra-legal measures that restrict peasants’ access to social goods (healthcare, education, and public infrastructure), land, and labor protections. These conditions result in what Shivji (2017) describes as people living “sub-human lives” while enduring “super-human labor.” Shivji echoes Edelman in highlighting the challenges faced by many impoverished people, who experience minimized consumption and maximized labor. He refers to this group as the “working people.” Shivji’s example from Tanzania provides further insight relevant to this case study. He explains how labor for mines and plantations was drawn from migratory labor reserves, akin to “indentured labor.” Traditional gendered divisions of labor ensure continued social reproduction and farm work in home villages, typically shouldered by women. This results in both peasantization and semi-proletarianization occurring within the same household, but in different locations, effectively subsidizing capital to generate “super-profits.” The two terms, “peasants” and “working people”, both highlights the precarious conditions faced by the rural populations when navigating through the impacts of land rush and the changing labour strategies.

## Methods

**Fig. 1:**
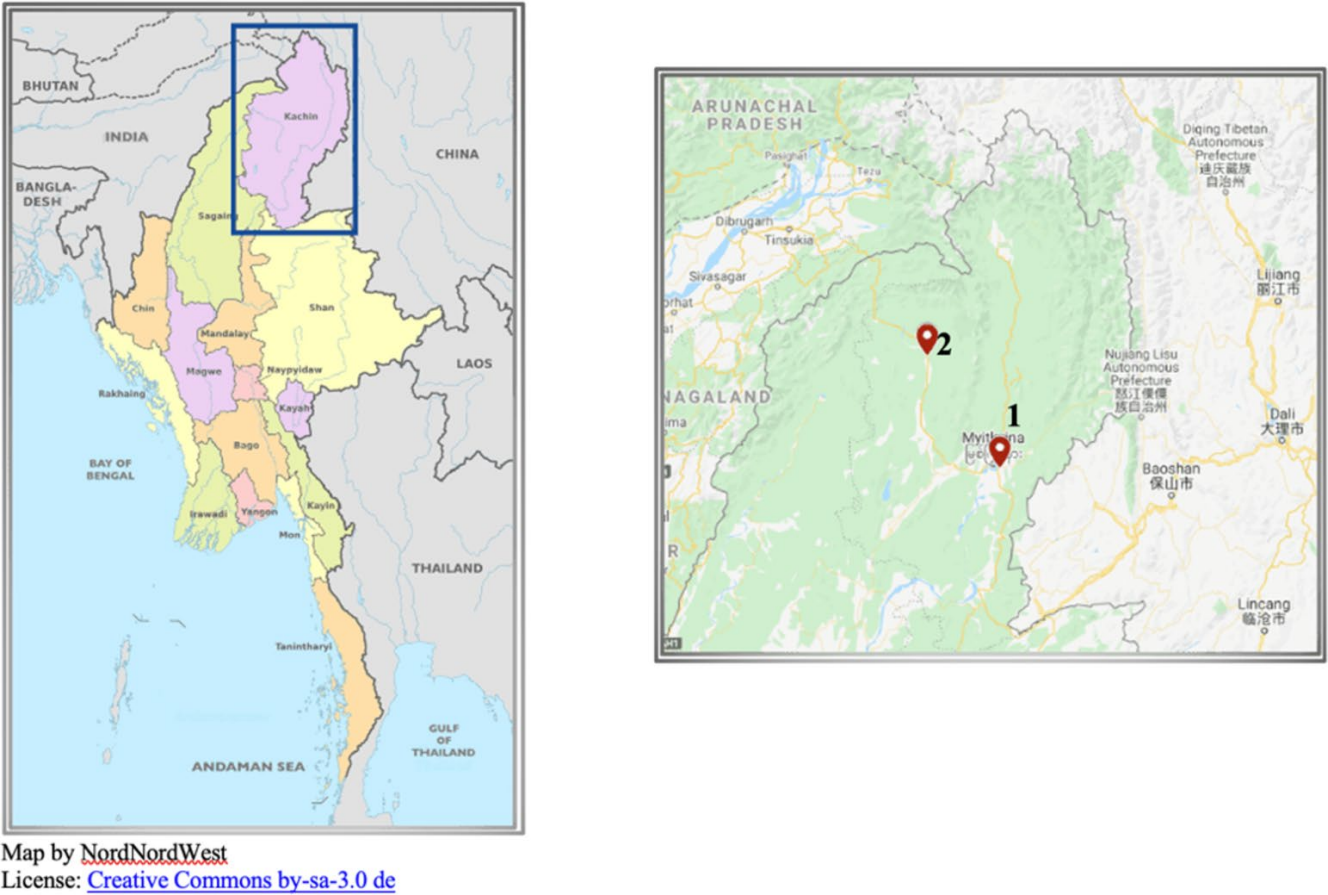
Location of Kachin State (in blue box) and Fig. 2 Primary field work sites (red markers)

**Fig. 2:**
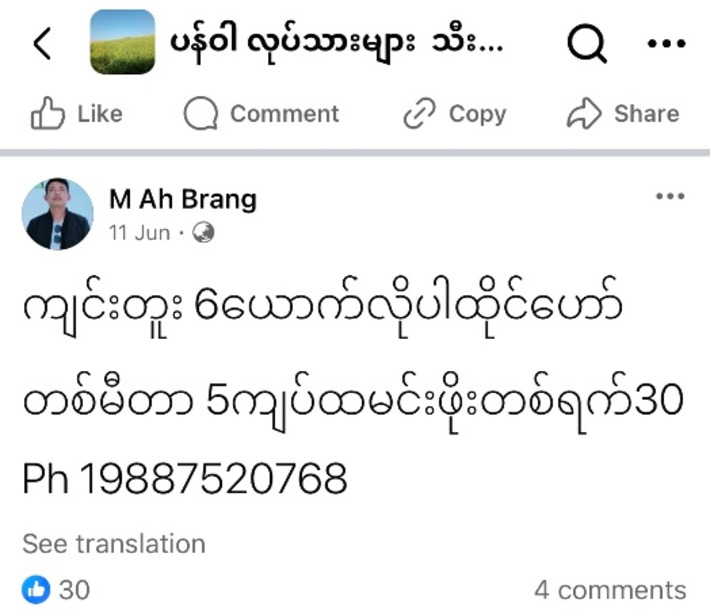
job advertisement on Facebook to work in the rare earth mines Source: (Brang 2024)

The study employed a mixed-method approach, incorporating both quantitative and qualitative data collection through household surveys, interviews, analysis of NGO and government reports, and monitoring social media sites such as Facebook. For the household survey, a purposive sampling was conducted in the selected villages in Kachin state as shown Figs. 1 and 2 below. Criteria for inclusion in the survey were households who are engaging in farming with or without the use of wage labour and depend on farm produce for family consumption or commercial selling. In particular, the criteria also included that the households had been affected by the land-rush induced land deals including biofuel land concession in Hpakant Township and the banana crop boom in Waingmaw Township. In December 2021, the first phase of the household survey was conducted with a total of 154 households in five villages in Waingmaw Township. Residents of two of those villages were displaced and living in various IDP camps. The second phase of the household survey, which included 68 households, was conducted in Hpakant Township in two villages and two resettled villages over five days in January 2022. The duration of data collection was intentionally shortened due to the extremely volatile political situation following the military coup in February 2021. At the time, it was believed that the military coup could lead to further armed clash between the state military and ethnic armed groups (as well as with the newly formed resistance groups) throughout the country. As a result, the surveys in Waingmaw and Hpakant townships were conducted as quickly as possible. The non-response rate of the household survey was low, except when the villagers were busy working on their farms or travelling; otherwise, respondents did not refuse to participate in the survey.

A total of 13 in-depth interviews were conducted in-person between 2022 and 2024 with nine individuals including leaders from the village, religious organization, ethnic armed organization (EAO), local development organization and National Unity Government of Myanmar (NUG). All of them were males which is an unintended circumstantial outcome, due to the inability to plan with the ongoing political instability and active armed conflict. Focus group discussions were carried out in Waingmaw township with two groups separately: IDPs and villagers, including five males and five females. Due to political sensitivity and the coincident timing with paddy harvest season, group gatherings were challenging in both townships. Hence, the researcher was only able to organize two sessions in Waingmaw township.

One thing to note is that the household surveys yielded valuable insights beyond strictly answers to the questions. Respondents provided contextual information to elaborate on their answers, and interviewers probed further as needed. Thus, many of the household surveys conducted became like individual in-depth interviews. To mitigate potential biases inherent in individual interviews and focus group discussion, data triangulation with findings from household surveys, national census data, and archival records was conducted to identify discrepancies and understand the significance of any systemic biases (Poggie 1972). Another important source of data was social media. Facebook as the most popular social networking in Myanmar has become an unlikely source of information (real time and historical), shared by individuals, social networks, and organizations, which of course needed further triangulation as mentioned above.

## Brief background on the study location

Located at the crossroads between China and India, Kachin State is one of the 14 states and regions established after the country’s independence from the British in 1947. It has an area of 89,041.8 square kilometers and a total population of 1,689,441 according to the 2014 census (Department of Population 2015). “Kachin” is the name of an ethnic group that can be further divided into six subgroups called Rawang, Lisu, Jinghpaw, Zaiwa, Lachik, and Lawngwaw. The Kachins, along with other ethnic groups such as the Shan and Bamar, have deep historical ties to the landscape. Kachins are also found across the border in China (Yunnan Province) and India (Arunachal Pradesh and Nagaland). Kachin State is also an economically strategic location due to its rich mineral resources (it is particularly known for jade, gold, amber, and rare earth minerals) and fertile soil for agriculture (notoriously associated with large-scale banana cultivation). At the same time, small- and medium-scale agriculture is predominant, growing a mix of subsistence and cash crops, including rice, peanuts, sesame, sugarcane, bananas, and corn. Traditionally, the Kachin practiced a customary land tenure system under chieftaincy and relied on subsistence-oriented shifting cultivation, supplemented by hunting, forest foraging, animal husbandry, and artisanal mining (Leach 1954).

Kachin State is also a landscape of armed conflict, suffering from more than 70 years of war between the state military and the Kachin Independence Organization (KIO). In 1961, over a decade after the country gained independence from the British, a new armed movement emerged in Kachin State led by Kachin World War II veterans who pledged to fight for “dimokrasi” (democracy) (Sadan 2013). This insurgency was primarily driven by ethnic and religious-based political oppression by the Bamar Buddhist majority central government, which came to power shortly after the country’s independence. Resource extraction, particularly jade, which has benefited Kachin chiefs and their clans, became central to the Kachin struggle for ethno-territorial and ethno-cultural rights. In its early years, the Kachin Independence Organization (KIO) traded raw jade blocks for weapons and ammunition from Chinese Nationalist (KMT) forces that had retreated across the border into Burma and were waging their own war against the Chinese Communist Party (Levy and Scott-Clark 2001). Although negotiations between the state and the KIO took place in the 1960s and 1970s, they ultimately failed. A splinter group of the KIO called the New Democratic Army-Kachin (NDA-K) signed a ceasefire agreement with the Myanmar government in 1989 in exchange for territorial control of a small border area within Kachin State called Special Region One. The region later became a hotspot for rare earth mining. The latest figure indicated that the war has displaced more than 130,000 people within the state and near the border with China (UNOCHA 2025).

### Enter the land rushes

In 1994, under internal and external pressure, the KIO decided to sign a ceasefire agreement with the military government. In a short period of time, the ceasefire agreement brought significant changes to Kachin State. As one land activist put it, the ceasefire period marked “ground zero” for an unprecedented land rush in Kachin State (IDI 09, personal communication, August 2022). On paper, the main goals of the agreement were stated as peaceful well-being, development, and economic growth (Maran 2015). However, the military began to deploy strategies to reproduce conditions of hegemony in the new ceasefire frontiers through a spectacle economy, as discussed by Tsing in the Indonesian context (2005a). Around the same time, the military government engaged in a dramatic act of investment prospecting, declaring over 22 million acres in Kachin State available for investment (MOALI 2000). This act was made possible by the revival of the Wasteland Instruction, a legacy of British colonialism, which allowed for the expropriation and reallocation of land classified as “wasteland,” a category that included land without land titles. The grant period could be for an initial period of 30 years, with the possibility of renewal for 10 years at a time, up to a total of 50 years.

This shift paved the way for the emergence of a range of projects in the ceasefire areas, particularly large-scale agricultural plantations, logging, and mining concessions (Buchanan et al. 2013; Kramer 2009, 2021; Woods 2011). Applying Tsing’s (2005b) analysis, the convergence and friction between the three components - frontier culture, franchise cronyism, and finance capital - of a spectacle economy opened up the Kachin landscape, with the central state as the key actor. In the Myanmar context, the situation is also referred to as “ceasefire capitalism” (Jones 2014), in which the central state granted land concessions in ceasefire zones as an explicit post-war military strategy to govern land and population with the aim of creating a regulated, legible, and militarized territory (Woods 2011, p. 747). The ceasefire-driven land rush was also further fueled by the drivers of the global land rush, which centered around crisis narratives of fuel, food, climate change, finance, and global governance (Borras Jr and Franco 2024; Zoomers 2010). The hype around flex crops such as jatropha (Borras et al., 2020), rubber (Global Witness 2015a), and corn (Woods 2020) has drastically affected land and labor dynamics. Zooming in on the study site of this paper, in 2006 a 200,000-acre land concession was granted to the Yuzana company for the large-scale production of biofuel crops (tapioca and cassava) (KDNG 2010).The catchy slogan of “national self-sufficiency” was used to justify the mega land concession to address the food and fuel crisis. This has significant implications for the reproduction of rural households and peasant agriculture, to be discussed below.

Beginning in 2011, a new political phase, dubbed “disciplined democracy” (Global Witness 2015a), emerged in Myanmar. This phase, part of the military’s political roadmap, retained significant military control through the state constitution. In 2011, a new electoral regime led by former military generals took power. In 2016, the National League for Democracy (NLD) government, led by Aung San Suu Kyi, came to power. The new quasi-civilian (or quasi-military) governments implemented neoliberal economic reforms with the full support of international institutions such as the World Bank, International Financial Corporation, Asian Development Bank and Japan International Corporation Agencies. The country came to be referred to as “the final frontier of Southeast Asia,” “Asia’s missing link,” or “a vast greenfield opportunity” with untapped natural resources and a consumer market of nearly 60 million people. State efforts at institutional reform to facilitate the commodification of land and facilitate corporate investment have been shaped by the military and other powerful actors representing the interests of capital classes (Jones 2014; Suhardiman et al. 2019). A barrage of laws, policies and other legal instruments from successive state regimes have been cemented in the institutional sphere, creating a stacked legal framework with overlapping claims from different government ministries and business sectors (Burma Environmental Working Group 2017). The ambiguity created by this situation has allowed powerful actors, in collusion with local authorities, to exploit the legitimate land claims of the poor (Mark 2016). Not surprisingly, a new land rush emerged, initiated by these actors “from above”, with the new regime acting as a facilitator. During this period, however, the dispossession of the rural population was further normalized and masked by the rhetoric of economic and political reform.

By early 2018, nearly 900,000 acres of agricultural concessions had been awarded in the Kachin State to agribusinesses (Department of Planning 2018). Driven by speculation and the spectacle making economy, the land rush often ended in hyperbolic projections and failed land deals as in the case of the rubber and jatropha boom-bust cycles of the early 2000s. Intensive logging by local and foreign companies in collusion with the military and ethnic armed groups was also one of the underlying reasons why three-quarters of Myanmar’s agricultural land concessions became unproductive and were labeled as “failed” land deals as they did not materialize as planned and were abandoned by the companies after logging has been completed (Borras et al. 2022). Previous studies on failed land deals have already suggested that the lands do not necessarily revert back to the rural working people and produce unsettling impacts on the affected communities in the short, medium and long terms (Borras et al. 2022a, b; Broegaard et al. 2022; Tarkapaw et al. 2016).

Land rush not only affected agricultural sector but gave rise to the mining rush embedded in the “politico-complex” emerged since the ceasefire period, involving the military, the KIO, local militias, and foreign and local capital (Jones 2014). Currently, the region produces 90% of the world’s most prized jade, generating annual revenue as high as US$30.9 billion in 2014 (Global Witness 2015b). This is equivalent to 47% of Myanmar’s total GDP in 2019 and one hundred times higher than the official government expenditure on public health, which was an estimated US$82.24 million in the same year (World Bank 2015). Kachin State is the only region in Myanmar where rare earth mineral (REM) mining is reported to be taking place. This REM rush in Kachin State reflects the global trend in energy transition from fossil-based energy to renewable energy sources to address climate change crisis. Since 2015, Myanmar has become a key supplier of heavy rare earth minerals to China (Qian 2021), providing 50% of the world’s dysprosium and terbium, two crucial elements used in high technology equipment (Xuanmin & Jun, 2021). Another mining rush taking off since the ceasefire period is gold mining, which has led to the intensification and expansion of gold mining activities throughout Kachin State (KDNG 2007). Gold mining affects most of the landscape in Kachin State, using varying levels of capital and technology (Myanmar Resource Watch 2023a). This ranges from small-scale, artisanal mining by individuals and families (although on a much smaller scale) to medium to large-scale operations using heavy machinery and migrant workers.

## Peasant farming or the “hanging-in” sector in Kachin state

The following case studies from Kachin State illustrate the situation of peasant and working people’s households in the context of the land rush. These cases highlight the diverse livelihood strategies and gendered and generational divisions of labour they employ to ensure daily social reproduction in the face of ongoing challenges. While these households share a common experience of land dispossession, political insecurity, and state neglect, their circumstances and adaptive strategies vary, reflecting the socially differentiated realities of rural life.

### Case #1

Hkawn Nu and her husband, both over 50 years old, live with their three sons, two daughters-in-law and one daughter. She has three grandchildren under the age of three. To her, land holds importance for multiple purposes such as land for housing, land for farming, land as food garden and livestock rearing space, and land for transfer to her children as inheritance. Before 2008, the family lived in their own house with a food garden, and engaged in farming full-time on a three-acre upland plot. In 2008, Yuzana company, with the approval from the state, confiscated their land for jatropha and cassava plantation project. As a result, they became homeless and landless at the same time. In return for their loss, they received around 450,000 Kyat (US$ 113)^2^ as compensation, which is significantly less compared to their loss. She still hopes that her family would be able to get the lands back.

Now her family is able to access 2 acres of upland for farming, which they bought from their relatives and grow rice for family consumption, one cycle per year. She raises some chickens for family consumption and pigs for selling at a local market, which is a common livelihood strategy among rural Kachin households. One of her sons and daughter work the land for half of the year without external labour. For farming, they have to spend 50,000 Kyat (US$ 12.5) to buy seeds from the local market, which is the only expense for farming as they do not need the use of farm machines or outside labour. They use family savings or took loans from others in case they need to spend on additional expenses. They also sell any surplus produce (less than half) to the local trader, who she reported to give a fair price sometimes.

In addition to farming, two of her sons work in both jade and gold mining in the same region, earning around 200,000 Kyat (US$ 50) per month in addition to occasional bonuses. The son who works in the jade mine does not receive a regular salary, as his income depends on the profit that can be made based on the quality of the jade that he and his team can collect. They send almost all of their migrant wages back home (80%), as the reason they work away is due to insufficient income at home. Their two sons only come back for two months a year during the rainy season, when mining has to be temporarily stopped and provide help on the family farm. Her family recently bought another motorbike to add to an existing one with money earned from gold mining. The household can earn about 500,000 Kyat (US$ 125) by selling surplus produce, 1,000,000 Kyat (US$ 250) by selling pigs, and 2,400,000 Kyat (US$ 600) from wage labour in one year. Hkawn Nu, as a mother and grandmother, takes care of household work while taking care of the home garden and domestic animals. They have never received any help from outside the family.

### Case #2

Naw Aung (32 years old) lives with his wife, son and younger brother. The village they now live in is called San Pya (“Model”) Village, where they were resettled after being evicted from their homes in their old village by Yuzana Company. As compensation, they were given a small plot of land for housing where his family currently lives. The family’s livelihood includes farming on two small upland plots that were not taken by the company because they are far from the village and the company’s activities. For Naw Aung, the land serves multiple purposes: farming for consumption and income, growing a food garden and raising chickens, and providing a playground for his son. The family rents farm machinery from a company using their savings, and occasionally borrows from private lenders to afford farm inputs. Their upland cultivation includes bamboo and djenkol trees. Recently, they informally gained access to an additional 4 acres of land abandoned by the Yuzana company after the KIO forcibly took control of the concession area in 2021.They chose to grow rice on the newly occupied land because of its agronomic viability, potential profit, and for family consumption. The couple works the land for half the year and employs three farm workers for weeding, planting, and harvesting. They pay the labourers a daily wage of 8,000 kyat (US$2) and need outside labour for about 15 days per season.

In search of a better life, Naw Aung’s brother migrated to Shaduzup village, which is a gold mining hotspot located not far from their own. He works in a goldsmith shop, earning 200,000 Kyat (US$ 50) a month and sends home 40% of his earnings. Naw Aung also works as a carpenter in his village and in nearby villages whenever work opportunities arise. He identifies carpentry as the family’s primary livelihood, followed by farming and wage work at a goldsmith shop They manage to save some income each year. Food is their biggest expense, follow by healthcare costs. The family struggles with access to health facilities, recreation spaces, and the internet for news updates. The military occasionally cuts off internet access in Hpakant Township whenever the war with the KIO intensifies. He said they sometimes can get help from the religious leaders or the local militia. The militia share control of the gold mining area with the military whose command it follows, and maintain a tense relationship with the KIO. *Social reproduction squeeze and labour dynamics*.

The two case studies illustrate the impact of the land rush phenomenon in Kachin State. Yuzana company’s acquisition of 200,000 acres for biofuel production, driven by the global rush for land and natural resources, directly resulted in the displacement and landlessness of Hkawn Nu’s family. This exemplifies the “spectacle-making economy” and “ceasefire capitalism” discussed earlier, where large-scale land concessions, often fueled by speculative investments and state-led initiatives, lead to dispossession and disruption of rural social life. On the other hand, Naw Aung’s family, while not directly dispossessed of their farmland, lost their homes and was forced to relocate to a new village, highlighting the social and spatial reorganization that often accompanies land rushes. Their subsequent access to abandoned land following the KIO’s intervention further demonstrates the volatility and contestation surrounding land access in this context. The land rush, with its boom-bust cycles and shifting power dynamics, creates enduring precarity on the ground. Moreover, both families’ engagement in wage labor, particularly in the mining sector, is a direct consequence of the land rush which has transformed the landscape and restructure local economy. While they struggle to meet the needs of daily social reproduction by selling their labour outside the farms, they also struggle to afford the labour costs on their own farms.

As the price of commodities continues to rise each year, the cost of agricultural labour has also increased, squeezing the profit margins of smallholder farmers like Hkawn Nu (case 1) and Naw Aung (case 2). These farmers, who cultivate small plots of land primarily for their own consumption and commercial sale of the surplus, face increasing challenges in securing affordable labour. They have to compete with larger commercial farms, mining and related businesses, largely controlled by capital, which can offer higher incomes and longer working hours. This competition for labour, coupled with repressive land and agricultural policies, raises concerns about the long-term viability of peasant agriculture in Kachin State. While only a few households have managed to accumulate land and engage in commercial rice production, many others are struggling to maintain their agricultural production. The diminishing availability of affordable farm labour, rising production costs and political volatility threaten to exacerbate the social reproduction squeeze. However, this situation also presents opportunities for farm workers, who now have greater leverage to demand better wages and choose from a wider range of work options.

The changing labour landscape is directly reflected in the costs associated with farming. Of the total costs of farming reported by farm households, labour costs accounted for 49%, the largest share of costs associated with farming, as shown in Table 1 below. Other significant costs included the cost of hiring farm machinery and the purchase of inputs such as herbicides and fertilisers. These costs are reported to be covered by household savings and no farming households reported receiving government subsidies. The lack of government support is also linked to the lack of formal land titles, reported by over 75% of households. As a result, they are unable to apply for government loans from public banks at low interest rates. Given these financial constraints, if a household works more than 3 acres of land, it is considered relatively better off than many other village households (IDI 02, personal communication, February 2022). Below is a typical cost matrix for a 4-acre paddy field as reported by a household survey respondent.

**Table 1:** Cost matrix of paddy cultivation on 4 acres of farmland as reported in January 2022

Item	Cost per unit (Kyat)	Total cost (Kyat)	Total cost (US$)
Tractor	25,000 per acre	100,000	25.00
Harvestor	5 baskets per 100 baskets	Depends on harvest	Depends on harvest
Land clearing	10 labourers for 2 days	140,000	35.00
Planting/broadcasting	15 labourers for 2 days	210,000	52.50
Harvesting	60,000 per acre	240,000	60.00
Herbicide	50,000 per two bottles	50,000	12.50
Bundling	4 labourers for 2 days	56,000	14.00
Seed	7,000 per one basket	35,000	8.75
Total cost incurred		831,000 (49% is for labour wage)	207.75
Total income from farming reported depending on the amount of surplus produce sold		Between 800,000 to 3,000,000 per year	Between 200 to 750 per year

*Daily wage rate range from 7000–8000 Kyat (US$ 1.75–2.00). Women usually earn less than men

### Struggles for social reproduction and labour fragmentation

In order to cope with the crisis of social reproduction, members of farming households combine different livelihood strategies to meet individual and household needs, as shown in Table 2 below. A clear pattern observed is the significant flow of rural-rural migration. Due to the pattern of “jobless growth” and the increasing flexibilisation of labour, securing a decent paying job in urban areas has become increasingly difficult for many rural people, which is also the case in Myanmar (Ossome 2024; Rodrik 2016). The high cost of living in the city also discourages people from seeking work in urban areas, as it affects their ability to send remittances back home. This brings us to the most important factor in people’s motivation to engage in migrant labour which is the income factor. Of the households citing a reason for migrant labor (*N* = 68), over 40% identified income as the primary driver. As the cost of living increases, it becomes more difficult for people to afford necessities such as food, non-food items and basic social services. According to the household survey, the top three categories of “major” expenditure reported are “food”, followed by “health and medical services” and “education, culture and recreation”.

The Table 2 below illustrates the wide range of activities undertaken by Kachin households, from agriculture and wage labour to handicrafts and petty trade. Aside from seasonal agricultural work, only the mining sector appears to offer a consistent demand for labour within Kachin State. This sector offers the flexibility and proximity that peasant households prefer. Often adults aged 18–49 divide their time between agriculture and other livelihoods such as mining. They prioritise work that offers flexibility, proximity to their villages and good income potential, particularly due to the need to return home during planting and harvesting seasons. Alternatively, adults also engage in long-term migrant work, sending remittances home and receiving farm products from home, especially rice and dried meat, to reduce food costs.

The mix of livelihood strategies has implications for the division of labor within households, often falling along gender and generational lines (Chazali et al. 2024; White 1989, 2012). Mothers, grandmothers, and daughters-in-law typically perform social reproductive work within the household, including home maintenance, child care, cooking, and tending to the food garden and domestic animals. In many households, women between the ages of 18 and 49 often contribute significantly to agricultural work, contributing similar labour time as their male household members. In this situation, women can consider to be shouldering “double work” across the spheres of economic production and social reproduction, helping to reproduce peasant households and peasant agriculture at the same time (Razavi 2009; Shah and Lerche 2020). Those over 49, typically grandparents, focus on productive tasks such as tending home gardens, raising animals, and caring for other household members. This caregiving role is essential, as it allows younger family members to dedicate their time to farming or other income-generating activities. Elders also play a vital role in the cultural reproduction of the village community, both in everyday life and during traditional events.

**Table 2:** Type of wage labour engaged in by peasant household members

Type of work engaged in by peasant household members	Duration	Remuneration
Seasonal farm work on other small/medium farms in rural areas	A few days to two weeks based on tasks and land size	Daily wage: 7000–10,000 Kyat (US$ 1.75–2.50) per day
Seasonal farm work on large-scale commercial farm in rural areas	A few weeks based on tasks	Daily wage: 7000–10,000 Kyat (US$ 1.75–2.50) per day
Migrant work in urban areas (car repair, grocery shop, tailor, carpenter, gambling center, school, marketing manager)	Usually long-term, although finding such work can be difficult due to limited availability and the need for specific skills	Depending on work starting from 100,000 Kyat (US$ 25) per month
Migrant work in other countries (destinations include China and Malaysia)	Can be seasonal and long term in China due to proximity (need connections to find work) Long term in Malaysia	Depending on work starting from around 500,000 Kyat (US$ 125) per month
Migrant work in the Mines (jade, gold, rare earth minerals)	A few months to a few years, relatively easy to find due to the constant need for physical labour	Gold mining– starting from 250,000 Kyat to 700,000 Kyat (US$ 62.50–175) per month depending on the tasks. Or has the option of dividing shares based on the gold yield Jade mining as jade scavengers - starting from 350,000 Kyat (US$ 87.50) per month. Irregular income. Divide share with the boss and other team members based on the quality of jade found. Rare earth mining– starting from 600,000 to 2,000,000 Kyat (US$ 150–500). It is a monthly wage work without additional or reduced renumeration.
Serve at Kachin Independence Organization/Army in rural areas	Long-term, available to adults and may or may not be voluntary	Starting from around 50,000 kyat (US$12.50) per month for basic positions. Payment is not regular and depends on the officer in charge.

## Unequal synergies between peasant farming and the mining sector

While peasant economy struggles, the mining sector in Kachin State has been booming. This growth has created an interdependence between the two sectors, offering migrant wage work for rural populations, while simultaneously exacerbating existing vulnerabilities for peasant communities, which has been described as the “double binds” situation (Lyall and Ruales 2025). At the same time, the intensified mining rush since the ceasefire period threatens both direct and indirect access to land for peasant households. This access is crucial not only for agricultural production but also for social reproduction, encompassing a wider range of activities that sustain livelihoods and communities. In the Kachin context, social reproduction extends to the right to exist as an ethnicity with its own territorial and cultural autonomy, a core point of contention between the KIO and the military.

### When the land is needed

When the household survey was conducted in the villages (including IDPs), the paddy land holding size of the households was explored in order to assess the “who owns what?” question as framed by Bernstein (2010) to understand the social relations of property in the villages. Of the 167 respondents, approximately 60% (100 respondents) are smallholders, defined in the Myanmar context as possessing less than 10 acres, and 20% (34 respondents) are landless. Some respondents own between 10.1 and 20 acres (7%), 20.1 to 50 acres (12%), and 51 acres or more (1%). Only around 30% of the households are reported to have land titles for all of their farmland plots. In contrast, individuals from outside the villages are reported to own more than 1,000 acres. Since the ceasefire agreement, the central state has allocated significantly large land concession to the corporations and individuals using the VFV Law and other legal instruments than officially reported. At the same time, land transfer to the more powerful actors take place through informal mechanisms, both in large-scale and in “pin-prick” forms (Borras Jr. et al., 2024). These conditions are described by a local land activist as followed:

Gold miners said they already pay tax to the officials. You can report to anyone you like. But we told them, officials “eat” the tax money. We peasants don’t take or eat any tax. You better get out from our village area. When we complained to the officials about the issue, they assured us not to panic and that those operations will stop possibly in December. But gold mining has not stopped. Instead, around 70 to 80 machineries came into our area to do gold digging along the Mogaung river. (IDI 06, personal communication, February 2024)

And another informant added,

Locals sell their lands to the rich people. The rich then pay to KIO to carry out gold mining on the newly acquired lands, polluting nearby river. Locals cannot protest because they are doing business on their own lands. We are told that impact on water source is concerned with the authority, not with the village. We cannot say anything. They also donate money to the church. (IDI 08, personal communication, February 2024).

This displacement also affects vulnerable groups like IDPs, who face double dispossession– first from their land due to war, and then again as mining operations encroach on their villages. One informant shared:

Villagers from Lawt Ja have to run flee because of war. But when they are unable to return, the companies started to mine gold in their villages by negotiating with the KIA, military, and militias. This is not fair for them at all. They have to rent land from others to farm. They haven’t received any compensation for their losses from anyone. Some children of IDPs end up working in shops, earning around 60,000–80,000 Kyat per month. The same situation is happening in the village north of Lawt Ja. They can’t return because of the gold mining operations that have destroy their village lands. Now, they also have to rent farmlands, where they grow sesame for selling. They are forced to start their lives anew. They are no more land to call their own. (IDI 03, personal communication, February 2022).

Returning to the household survey findings, we see more than half of peasants have access to less than ten acres of land while 20% of them are landless. However, the dataset, if used alone, adopts a production-centric lens, focusing primarily on land used for agriculture. This perspective obscures a crucial aspect of land access in Kachin State: the increasing expropriation of land traditionally used for social reproduction: forests, rivers, communal spaces, common grazing grounds, and even sacred places have been subsumed by mining, commercial agriculture, and conservation projects. By overlooking these forms of land dispossession and expropriation, the data fails to capture the full extent of land rushes that intensified in Kachin State after the 1994 ceasefire and again with the 2011 political transition.

The quotes above also highlight how mining operations not only draw labor from peasant households but also “draw” land (including rivers and forests) away from them, restricting or completely cutting off their access to the landscape. One village elder from Lawt Ja who can no longer return to his village bitterly bemoaned, “If my head contains traces of gold, they might even dig into my head” (WM IDP). On the other hand, many villagers have been forced to sell off their lands as the surrounding landscape becomes unlivable due to mining activities. As the land accessibility and livability become exponentially shrunk, the villagers are forced to resort to migrant work, most often in the mining sites away from their homes.

### When the labour is needed

Mining hotspots have become increasingly popular work destinations, particularly among the younger generation from peasant households where they sell their labour under precarious conditions. While wages are often low, they can still be relatively higher than those in agriculture. This allows them to contribute to their families’ income while maintaining a connection to their land and rural livelihoods. Their ability to sell their labor power at a cheap rate is made possible by their access to land for farming and for social reproduction activities in their villages, as well as income contributed from other household members’ wage work. In return, a portion of their wages from mining work is invested in farming and the social reproduction of their households. This movement of labor between agriculture and mining creates a symbiotic, albeit unequal, relationship. Peasant farming, which sustains the land and provides food for the people, and the extractive mining sector, which often depletes it, become intertwined. A local development worker eloquently captures this entanglement:

Before in the old days in our areas, they grow rice in rainy season. On the uplands, it would start from May and on flat farmlands, it begins from June and July. The produce, for example rice, some portion they keep for consumption and a small portion is reserved as seeds for the next season. And then the rest they sell. Year by year, the context has changed. They have to earn more income. So, they end up selling more of their harvest. What I want to say is the paddy they grow in one year cannot cover them until the next season. So, when it is time for cultivation season, they are already in hardship. Since the old days, after the cultivation season, when the weather becomes drier, the villagers go to the mining site. What I mean with mining is the artisanal mining by hand in the rivers and streams nearby. Some would go to mining and some engage in wage work on nearby farms. It is because of the growing commodity price that they are compelled to do this. It is not enough to feed and take care of the whole households including to pay for school expenses, and so on. What is becoming more difficult at this time is the higher price of agriculture inputs. They cannot cultivate on the same scale as before. So, the consequence is that paddy produce is insufficient to cover for the next year. They now face longer duration of crisis. The current trend is people are going to the rare earth mines. (IDI 13, personal communication, 25 June 2024)

The ongoing armed conflict in Kachin State further complicates this relationship. Displacement and insecurity disrupt agricultural production, pushing more people towards the mines. This creates a vicious cycle where conflict exacerbates the very conditions that drive people to seek work in the mines. The same development worker expresses his concerns with regards to the impact of ongoing war that further disrupts food access, as the development worker explains:

We’re also facing a war that has displaced a largest number of rural populations. People cannot grow food in many areas due to the security concerns. This has created a surplus of labour, who end up seeking for work in the mining industry. I worry about our food security because unlike before, we’re in an extremely difficult situation. The more people who can’t farm, the more will turn to mining. It’s a snowball effect. (IDI 13, personal communication, 25 June 2024).

To sum up, the situation highlights the crucial importance of land and labour in Kachin State but demonstrates that the terms of their subsumption are most often dictated by capital, war, and power dynamics—essential animators of land rushes.

### Life in the mines

Like past mining rushes around the world, mining in Kachin State appears to offer the allure of “making instant capitalists, by erasing lines of class, and by making the free labour dream real”, as observed by Demuth (2020, p. 257) in her research on the Nome Gold Rush in Alaska. The allure of instant riches draws thousands of men and women in their prime to Kachin State from across Myanmar. The allure is further fueled by the hype around mine work which is promoted through songs, movies, and personal accounts. The most popular song lyric about the miners’ hard, yet ambitious life is expressed as “every miner is a ‘lao ban’ in the making” (lao ban comes from the Chinese word for boss), encapsulating aspiration for upward mobility.

However, the reality of mining is often harsh. Borrowing Sadan’s (2013, p. 97) words as she described the situation of jade mines in Kachin State, the mines are “a place of hard and painful work, temporary windfalls and great temptations”. While some are able to accumulate massive wealth, there are also parallel narratives with stories of those who faced personal and financial ruins in the mines due to drug abuse, gambling, or the inability to find high value stones (Levy and Scott-Clark 2001; Prasse-Freeman 2021; Sadan 2013). Nevertheless, the possibility of improving one’s socio-economic position, however slim, compels people like those in Hkawn Nu and Naw Aung’s households, to risk their lives to support a basic living —something peasant agriculture struggles to provide in the face of dispossession, expropriation, and oppression.

**Fig. 3:**
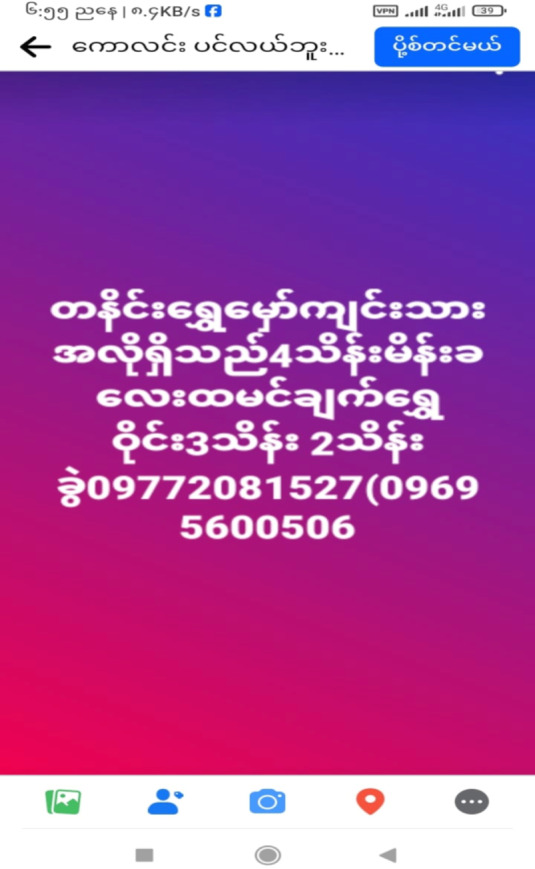
job advertisement on Facebook to work in a gold mine Source: (Moe 2024)

The destinations reported by rural households during the household survey for migrant work include Hpakant, Sadung, Pang Wa, Danai, Putao, Myitkyina, and Shaduzup– all of which are boom towns associated with mining of jade, rare earth minerals, and gold. A survey respondent provides a succinct summary of his job task description, stating, “Find jade, find gold, or sell things”, flexing his labour power at places where he can make good income. The demand for labour in the mines is evident in the numerous job advertisements found on social media platforms like Facebook (see Figs. 2 and 3 for two examples). The image on the right side of Fig. 3 reads as followed: “Gold miners in Danai wanted. 400,000 Kyat (US$100) for woman cook and 200,000 (US$50) to 300,000 (US$75) kyat for gold panner” (Moe 2024). Similarly, advertisements for rare earth mining jobs can also be found on social media, as illustrated on the left (Fig. 2): “6 miners wanted. 1 meter (5 yuan). Food allowance per day (30 yuan)” (Brang 2024). Work in the rare earth mines offer salaries of up to 2,000,000 Kyat (US$500) per month, making them highly attractive to Myanmar’s impoverished working people (Myanmar Resource Watch 2023b).

### Life in the mines

Many research studies describe the working conditions of miners as similar to what Shivji (2017) termed “sub-human” and “super-worker.” Miners work long hours, typically more than five days a week and exceeding eight hours per day, handling small but hard-laboured operations alongside giant excavators and machines. For the jade scavengers, their working hours typically extend from late evening to the next dawn, as they can only access the company’s land waste heap outside of the company’s operation hours. They have only small torchlights to guide their way amidst the ocean of rubble with a stave and a bag in the race against hundreds of other scavengers to find a valuable stone of jade. Nearly half of migrant miners send a significant portion of their earnings back home to their families, with an average remittance of 1,947,000 Kyat (approximately 430 USD) in the past year (Lin et al. 2019). Despite this, many miners struggle financially, with 25% reporting being in debt. This finding is also reflected in the household survey conducted where 30% of peasant households with a member engaging in mining work reported to have debt (ibid.).

The mining work is inherently dangerous, with landslides a constant threat, particularly at jade and rare earth mining sites. These hazards are becoming more frequent as a research study conducted on jade scavengers reported an estimated 500 deaths from landslides in the past year (Lin et al. 2019). The most notable accident occurred in July 2020, when a massive wave of mud and water caused by heavy rain killed approximately 170 jade scavengers, mostly youth (Lin et al. 2021). These tragedies demonstrate the high-risk conditions in which miners live and work. Many are housed in make-shift temporary shelters, sometimes dangerously close to the mines. Fatalities from landslides occur most often among those living near the mines. Until now, no mine owner or local authorities have been held accountable for the preventable tragedies.

Furthermore, exposure to dangerous chemicals is a serious risk in gold and rare earth mines. Concentrations of mercury, lead, cadmium, and manganese associating with gold mining have been reported in the N-mai, Mali, and Irrawaddy rivers (Myanmar Resource Watch 2024). Moreover, the heavy use of ammonium sulphate in in-situ leaching to unearth rare earth minerals, which locations are also close to N-mai river (Myanmar Resource Watch 2023b). These chemicals are immediately exposed to the mine workers and can cause serious health problems, including skin diseases, respiratory and gastrointestinal problems, and even cancer. Nevertheless, workers are provided with minimal or no protective equipment, increasing their risk of injuries and illnesses. Drug use, particularly heroine and methamphetamine tablets, is highly prevalent among the workers particularly in the jade hotspots where drugs are sold cheaply and very much accessible (Levy and Scott-Clark 2001; Lin et al. 2019). One of the reasons for taking drugs include to improve stamina and survive in the harsh working conditions.

Many women also accompany their husbands or come on their own for migrant work in the mines. The women may work alongside their husbands or engage in petty trades, but women are usually paid lower wages. At the same time, they engage in social reproduction tasks such as childcare and cooking, as documented by the Kachin Development Networking Group (KDNG) (2007). In addition, women face sexual exploitation by more powerful actors, where they are coerced into sexual engagement with bosses or managers to keep their jobs. In the rare earth mines, women are sometimes recruited specifically for this type of work (Myanmar Resource Watch 2023b). However, they lack access to proper healthcare to get treatment for sexually transmitted diseases or for unwanted pregnancies.

## Implications on the landscape formation

The recent land rushes in Kachin State, also linked to global processes and characterized by a chaotic and competitive scramble for land, have had profound impacts on the lives of peasant households and the formation of landscape. As this study has shown, the land rushes have resulted in the displacement of peasant farmers, loss of land access, and the expansion of extractive industries like mining. Land rushes have subsumed lands which are not only used by the rural communities for production but also for social reproduction, including farmlands, house plots, communal grazing ground, forest, and rivers. This has led to increased precarity for peasant households, forcing them to rely on labour fragmentation strategies, such as migrating for work in the mining and agribusiness sectors, to ensure their daily survival. The influx of cheap labour from rural peasant households in Kachin State and across Myanmar has fueled the growth of the mining sector. In turn, wages earned from these industries have provided a crucial lifeline for many impoverished rural peasant households facing social reproduction crises.

When taking a cautious look at the migration pattern, it reveals an entanglement between the two unlikely sectors, as peasant farming and mining industries become linked in the struggle for social reproduction. Peasant farming, with its “significant contribution to the maintenance of natural resources (soil productivity, landscapes, water, biodiversity, carbon-capture, and so on),” is a key pillar of food sovereignty (Van Der Ploeg 2014, p. 1019), which is also increasingly posed an alternative system to erode capitalism. Using minimal chemical inputs, primarily for self-consumption, peasant farming helps mitigate climate change and environmental degradation, effectively “cooling the earth” (Martinez-Alier 2011; Via Campesina 2007). On the other hand, the mining industry is associated with a destructive, non-reproductive relationship with the land, contributing to the ecological crisis as an intrinsic tendency of capitalism (Fraser 2021). It essentially “heats up the earth” (Norgate and Haque 2010). Despite the different character of the two sectors, both of them are symbiotically linked in Kachin State. Patterns of rural labour flow reveal the mining industry and peasant farming together prop up rural households as the former provides wage income, the latter provides a source of cheap and “super-workers” (Shivji 2017). This form of “smallholder slot,” as conceptualized by Peluso (2016), has emerged from a variety of historical, institutional, and structural factors that have shaped Kachin society.

One facilitating factor is the emergence of state mechanisms that have redefined land as property and reshaped property relations through laws like the the Wasteland Act and its later version, the Vacant, Fallow and Virgin Lands Management Law. These, along with the state’s short and long term spectacle-making economic plans driven by “ceasefire capitalism” (Woods 2011) have historically been biased against the peasant economy. The right to a customary tenure system and the right of the peasant households to derive benefits from the commons have been subsumed under a property regime that privileges individuals or corporations to their exclusive benefits (Macpherson 1978). This new form of property relations is then protected by legal or extra-legal force in the form of criminal codes or military violence. In Kachin State, the struggle over ethno-territorial rights, in the form of armed conflict or ceasefire agreement between the state and the Kachin Independence Organization (KIO), has produced significant constraints in peasant agriculture through displacement, and debilitating conditions for production and mobility. Amidst this violent and volatile environment, both the state and the KIO (and to a lesser extent, state-backed militias) have become key players in mediating property relations, both formally and informally. This has been witnessed in the state and the KIO approving formal and informal land deals to those with capital in exchange for rentier income, rather than protecting land access by the peasants and working people.

Additionally, the changing structure of Kachin’s rural economy, increasingly defined by mining and agribusiness, has negatively impacted peasant agriculture, as has been shown by the case of peasants in Nigeria with the oil economy (Watts 2013). The landscape has turned into patches of mining zones and monoculture plantations, where wealth accumulation by powerful actors comes at the expense of local ecology and social life. State policies and subsidies favor these capitalist enterprises, facilitating the transfer of cheap land and labour. As a result, the peasant agriculture has reached a stage of barely “hanging in” (Hall et al. 2017) with household members having to resort to a variety of livelihood strategies to address social reproduction crisis, where women are found to shoulder double work and the youth engaging in high-risk mining work. Using Mitchell’s (1996) words, the current landscape looks natural when in fact, it is not; instead “the land is now imbued with power and money”, as one key informant described (IDI 05, personal communication, February 2024, p. 05).

The penetration and expansion of market mechanisms also played an important role. This has been particularly evident with the rising commodity prices in the past, which escalated further during the pandemic and after the military coup. The increased costs of farm inputs such as herbicides, fuel, labour, and farm machinery have made farming less profitable than before. This economic pressure has driven many farmers to seek alternative sources of income, including mining, and pushes them to grow food primarily for consumption. This ensures access to food in the midst of rising costs and political instability. From the view of a neoliberal institution such as the Asian Development Bank, Myanmar’s agriculture sector is characterized as being in the “low equilibrium trap”, that is the smallholder majority farming sector is trapped in low input, low productivity, low quality output, and low returns, contributing to chronic poverty and food insecurity (Raitzer et al. 2015). While the analysis more or less reflects the current situation, they neglect to link the dire conditions to the underlying conditions and power relations. The evolution of rural peasantry into such a state of despair has been described by Watts (2013) as the “silent violence” in his vivid account of the Nigerian peasantry facing social reproduction crisis amidst the oil wealth. Specifically, he chose this term to highlight the structural factors and “the absences and neglect” from state, market, and power relations that caused the chronic agrarian crisis.

On the same landscape, a different form of market dynamic is playing out. The mining industry offers higher wages than small and medium farms, attracting labourers from peasant and working-people households who lack other options. Commodities such as jade, gold and rare earth minerals garner higher selling prices in the market; hence, the mine owners or bosses can pay relatively higher wages than the small and medium farms. But in comparison to the enormous profit that can be accumulated from the mining enterprises, the wages offered in the mines are very low. Yet the only requirements for the mine work are physical strength andadaptability to high risk working conditions, which make it very attractive and accessible to the young working force. The resulting impacts are increasing scarcity of seasonal farm workers and the driving up of agricultural wages, which further squeezes the income of peasant households.

Coming back to Oya’s question, “is land everything to the poor?“, my research in Kachin State provides a nuanced perspective. Amidst the violent context, peasant farming provides the primary means of subsistence for consumption and for commercial selling which enable the daily reproduction of peasant households. Without access to land, many would face even greater hardship (Ossome 2024). At the same time, access alone is not enough as has been shown by the Kachin case. It must be accompanied by redistributive reforms which focus on improving productive forces, living and working conditions, and access to state subsidies (Rigg 2006; Van Der Ploeg 2023). The current state policies deliberately structure the recognition of land rights and the right to benefit from land in a way that favor a few and disadvantage the majority. This leads to further differentiation within the peasantry, with some falling deeper into debt and precarity, while others manage to “dig in”– that is through self-exploitation of household labour power to maintain similar socio-economic conditions (Bernstein 2006; Scott 1976; Shanin 1972).

## Conclusion

In conclusion, tracing labour relations and social reproduction struggles in peasant households help to analyze entanglements around the rural agrarian sector, in this case, those who straddle between peasant farming and the mining sector. This also brings to mind the agrarian question brought up by Chayanov (1986), concerning the underdevelopment of the rural. This underdevelopment can be linked to the oppression of peasants as a class, and in the case of Kachin state, of people as an ethnicity. In her reflection on E.P. Thompson’s *Whigs and Hunters*, Peluso (2017, p. 313) wrote.

… but conflicting land uses, contentious power relations and property rights are the mainstay of agrarian capitalist relations then and now: wringing blood and life-force from whatever social relations exist on the land prior to their arrival. Not without a fight however.

It is exactly what is being witnessed in Kachin State. Kachin’s rural working people “make live” (Li 2010) under an immiserizing growth economy that compels them to the mines, knowing the resulting environmental impact. The rapid expansion of the mining sector has drowned out other development possibilities as the working people struggle to share the wealth produced from mining in the forms of petty wages and rare windfall, which can then flow to the peasant households. When wealth generated from the two sectors hugely differs, there is a serious need to rethink proposals around agrarian reform that center on redistributing land but not wealth (Rigg 2006). The case of Kachin State demonstrates that advocating for agroecology and food sovereignty requires a deeper probing around the political economy of peasant agriculture and peasant households. The processes driving the mining economy are intertwined with and, in some ways, support the persistence of peasant agriculture. This means that the mining industry, while is often viewed as detrimental to rural livelihoods, is paradoxically contributing to the survival of peasant households.

## Footnotes

1 In the context of this paper, peasants are a diverse group of people engaging in farming in combination with various livelihoods including wage labour, livestock production, artisanal mining, fisheries, forest foraging, petty trade, and petty commerce, and other skilled and unskilled occupations. Further elaboration will follow in the theoretical section.

2 Exchange rates in this study are based on the current market average of 1 USD = 4,000 MMK (Myanmar Kyat), unless otherwise specified.
